# Developing Successful Intelligence in Global Academia: A Triarchic Framework for EAP Pedagogy

**DOI:** 10.3390/jintelligence13110134

**Published:** 2025-10-23

**Authors:** Yang Yu, Yingying Xu, Yongkang Wu

**Affiliations:** School of Foreign Languages, Dalian Maritime University, Dalian 116026, China; xuyingying@dlmu.edu.cn (Y.X.); kk191@dlmu.edu.cn (Y.W.)

**Keywords:** Triarchic Theory of Intelligence, successful intelligence, English for Academic Purposes (EAP), practical intelligence, creative intelligence, academic literacies, sociocultural theory

## Abstract

This review synthesizes research from cognitive psychology and English for Academic Purposes (EAP) to propose a new conceptual framework for understanding and fostering international student success. It argues that traditional EAP approaches, while effective in developing analytical intelligence—evidenced by a focus on critical reading, argumentation, and source-based writing—provide an incomplete model for the multifaceted demands of global academia. Drawing on Robert Sternberg’s Triarchic Theory of Intelligence, this paper posits that “successful intelligence,” defined as the capacity to achieve one’s goals within a specific sociocultural context, is a more holistic and ecologically valid construct. It depends equally on creative intelligence (e.g., formulating novel research ideas, adapting to unfamiliar academic genres) and practical intelligence (e.g., navigating academic norms, acquiring tacit knowledge, demonstrating pragmatic competence in communication). This paper conducts a critical review of pedagogical practices within EAP that implicitly or explicitly cultivate these three interdependent intelligences. After providing a balanced overview of Sternberg’s theory, including its scholarly critiques, this review broadens its theoretical lens to incorporate complementary perspectives from sociocultural approaches to academic literacies. It systematically maps specific EAP tasks—such as source-based synthesis essays (analytical), research proposals for occluded genres (creative), and simulations of academic email communication (practical)—onto the components of the triarchic model. Drawing on this analysis, the paper concludes by proposing an integrated pedagogical framework, the “Triarchic EAP Model.” This model consciously balances the development of analytical, creative, and practical abilities through integrated tasks, explicit scaffolding, and a focus on transferability. It offers a more holistic approach to student support and strategically positions the EAP classroom as a unique environment for the cultivation and assessment of the multifaceted intellectual skills required for sustainable success in 21st-century global academia.

## 1. Introduction

### 1.1. The Changing Landscape of International Higher Education

The 21st-century higher education landscape is characterized by an unprecedented scale of student mobility, fundamentally reshaping institutional demographics and pedagogical priorities. Annually, millions of students pursue tertiary education outside their home countries, motivated by the pursuit of specialized knowledge, the desire for enhanced global competencies, and the promise of improved long-term career prospects. This global migration of academic talent injects substantial economic, cultural, and intellectual capital into host institutions, fostering diverse learning environments and catalyzing international research collaborations (de Wit and Altbach 2021; Yang 2022).

However, for the individual student, this transition represents a period of profound adjustment fraught with challenges that extend far beyond linguistic proficiency (Sheng et al. 2022; Wei 2025). A comprehensive body of literature documents the complex array of stressors that international students confront across academic, sociocultural, and personal domains as they navigate unfamiliar educational systems and cultural norms (Alharbi and Smith 2018; Ng et al. 2017). The process of adaptation is deeply multifaceted. It encompasses not only academic integration—mastering new pedagogical styles, assessment criteria, and expectations for critical argumentation—but also the development of sociocultural competence essential for managing daily life, building social networks, and interpreting implicit behavioral cues in new cultural milieux (Mao 2024; Searle and Ward 1990). Indeed, research conducted in non-Western contexts, such as China, highlights the unique sociocultural and academic adaptation challenges students face, underscoring the global nature of this phenomenon (Mao 2024; Wei 2025).

The failure to adapt effectively can precipitate significant psychological distress, including heightened anxiety, depression, and social isolation, which in turn severely impedes academic progress and overall well-being (Maharaj et al. 2024; Luo et al. 2024; Yin et al. 2024). Research consistently demonstrates a strong positive correlation between successful sociocultural adaptation and key outcomes such as student retention, academic performance, and psychological health (Glass and Westmont 2014; Razgulin et al. 2023). This evidence strongly suggests that academic success is inextricably linked to a student’s ability to integrate into the broader social fabric of the institution.

This reality underscores a critical need for proactive, integrated institutional support systems that move beyond a deficit-oriented, remedial view of language instruction. The imperative is to foster comprehensive acculturation and empower students to thrive, not merely survive, in their new academic ecosystems (Gu et al. 2010; Ryan 2011). This paper posits that this necessitates a fundamental reconceptualization of “academic readiness,” shifting the focus from narrow, measurable linguistic scores (e.g., TOEFL, IELTS) to a more ecologically valid and holistic construct of competence—one that prepares students for the full spectrum of intellectual and social demands they will inevitably face.

### 1.2. Limitations of Traditional EAP and the Analytical Bias

Within this institutional support network, English for Academic Purposes (EAP) programs serve as a primary conduit for the academic and cultural acculturation of international students. Over the past several decades, EAP has evolved from a nascent focus on linguistic accuracy to a sophisticated engagement with academic skills and discourse conventions (Cao and Hu 2024). Contemporary EAP pedagogy has achieved considerable success in developing the linguistic and academic competencies deemed essential for university study, such as critical reading, source-based writing, and oral presentation skills (Kalsum et al. 2023). The pedagogical emphasis is frequently placed on integrated writing tasks that require students to read, synthesize, and critically evaluate multiple sources to construct a coherent, evidence-based academic argument (Hyland and Jiang 2021; McCray and Hanks 2023).

While these skills are undeniably fundamental, this conventional approach implicitly and, at times, explicitly prioritizes the analytical dimension of academic competence (Sternberg 2003). This analytical bias, though valuable, provides students with an incomplete toolkit for navigating the full range of challenges they will inevitably encounter. Sustainable success in contemporary global academia demands more than the ability to deconstruct and analyze existing texts; it requires the capacity to generate novel ideas, solve complex practical problems, and navigate intricate social and institutional structures with acumen and confidence (Jackson 2015; Sternberg 2021b). The traditional EAP model, therefore, often falls short of preparing students for the multifaceted reality of becoming a successful and integrated member of a new academic community. It risks producing students who can write a technically proficient essay but who struggle to formulate an original research topic, manage a difficult conversation with a supervisor, or interpret the unspoken expectations of their disciplinary culture. The recent emergence of powerful generative artificial intelligence (AI) tools adds another layer of complexity, creating new challenges for academic integrity and the development of authentic student voice that traditional models are not yet equipped to address (Ros and Samuel 2024; Cotton et al. 2024).

### 1.3. A Case for Successful Intelligence in Academia

To address this pedagogical gap, a more comprehensive and ecologically valid theoretical framework is required. This review argues that Robert Sternberg’s (1985, 1997) Triarchic Theory of Intelligence, and its superordinate concept of “successful intelligence,” offers a powerful model for reconceptualizing the competencies required for academic success. This theory posits that effective functioning in any environment is not dictated by a single, monolithic entity (often narrowly equated with IQ) but by a dynamic balance of three distinct yet interrelated components: analytical, creative, and practical intelligence (Sternberg 2011). This paper contends that by adopting this triarchic framework, EAP programs have the potential to more effectively prepare students not only to meet the rigorous analytical demands of their coursework but also to navigate the complex creative and practical challenges inherent in becoming successful members of the global academic community. This approach reframes the goal of EAP from linguistic remediation to the cultivation of a holistic intelligence that is adaptive, goal-oriented, and context-sensitive.

### 1.4. Purpose and Structure of the Review

The purpose of this review is to construct a theoretical bridge between Sternberg’s psychological model of successful intelligence and the applied field of EAP pedagogy. It is a conceptual paper that aims to critique the analytical bias of current EAP practices and propose a more balanced, integrated model that addresses the full spectrum of intellectual abilities needed for international student success. The paper will proceed as follows. Section 2 provides a detailed overview and critical examination of the conceptual architecture of successful intelligence, including its evolution into a theory of adaptive intelligence. Section 3 broadens the theoretical lens by introducing complementary perspectives from other theories of intelligence and learning. Section 4 situates these theories within the context of EAP, offering a critical synthesis of current pedagogical practices. Section 5 leverages this analysis to propose the “Triarchic EAP Model,” an integrated framework for curriculum design and assessment. Finally, Section 6 discusses the broader implications, challenges, and future directions of this model and outlines a robust agenda for future research.

## 2. Theoretical Foundation: A Critical Examination of Successful Intelligence

A robust theoretical architecture is essential for constructing a more holistic pedagogy. Sternberg’s theory provides an evidence-based foundation for moving beyond narrow, decontextualized conceptions of academic ability and toward a model that accounts for the real-world demands of academic life.

### 2.1. The Triarchic Components: Analytical, Creative, and Practical Intelligence

Sternberg’s (1985) Triarchic Theory of Intelligence is grounded in three constituent sub-theories that describe the relationship between intelligence and the internal world of the individual, the role of experience, and its functional connection to the external world.

The componential sub-theory explains the mechanisms of analytical intelligence, specifying the mental processes (“components”) that underpin intelligent behavior. It delineates three types of components. Metacomponents are higher-order executive processes used for planning, monitoring, and evaluating problem-solving (e.g., recognizing a problem’s existence, defining its nature, allocating cognitive resources, and monitoring one’s solution strategy). Performance components are the lower-order processes that execute the metacomponents’ instructions (e.g., inferring relationships between ideas, applying logical rules to construct an argument, comparing and contrasting concepts). Finally, knowledge-acquisition components are involved in learning new information (e.g., selective encoding to distinguish relevant from irrelevant information, selective combination to integrate disparate pieces of data into a coherent whole, and selective comparison to relate new information to existing knowledge schemata) (Sternberg 1985, 2011). EAP instruction in critical, source-based writing directly engages all three: a student must plan their essay (meta), infer an author’s stance and apply principles of argumentation (performance), and connect ideas from multiple sources to build new understanding (knowledge-acquisition).

The experiential sub-theory elucidates creative intelligence by highlighting the dual ability to deal with novelty and to automate information processing. An individual’s capacity to handle novel tasks, problems, and situations is a key indicator of intelligence. This involves seeing old problems in new ways and challenging conventional wisdom. Equally important is the ability to take a once-novel task and, through practice, render its processing routine and automatic. This automation frees up finite cognitive resources to be allocated to new and more complex challenges (Sternberg 1997). This sub-theory is highly salient for international students, who are constantly confronted with novel academic genres (e.g., the literature review, the research proposal), unfamiliar communicative tasks (e.g., participating in a seminar), and new sociocultural expectations. The goal of education, from this perspective, is to help students develop the expertise to a point where they can navigate familiar tasks efficiently, thereby saving their cognitive energy for true innovation.

**Figure 1:**
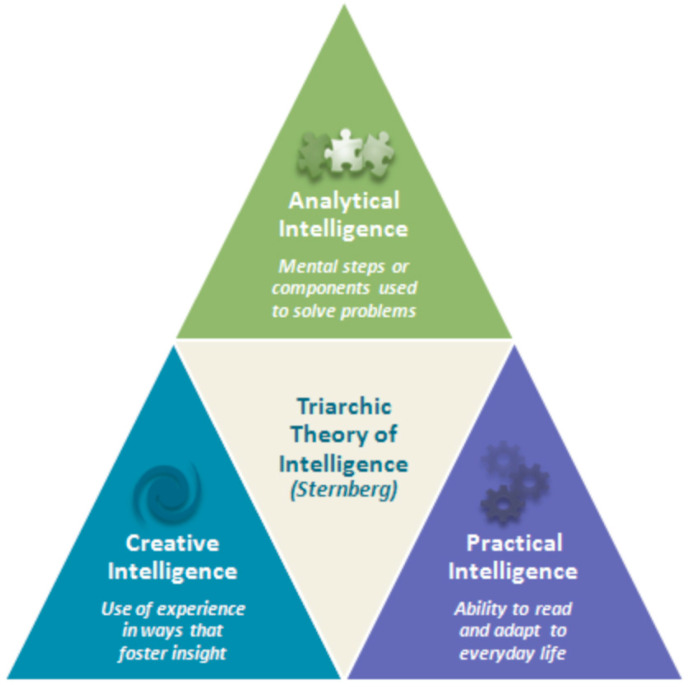
The Triarchic Theory of Intelligence (Sternberg 1985).

The contextual sub-theory defines practical intelligence as the ability to function effectively in one’s environment. It posits that intelligent behavior involves a purposive adaptation to one’s existing environment, the shaping of that environment to better suit one’s needs, or the selection of a new environment altogether. This subtheory emphasizes that intelligence is culturally and contextually relative; what is considered intelligent or effective behavior in one context may not be in another (Sternberg et al. 2000). Practical intelligence is largely procedural and acquired through experience, manifesting as “tacit knowledge”—the unspoken rules and “how-to” knowledge needed to succeed (Wagner and Sternberg 1987). For an international student, this could manifest as adapting to a professor’s preferred communication style (adaptation), proactively shaping a study group to be more productive (shaping), or selecting a research lab that is a better fit for their academic and career goals (selection). (See Figure 1).

### 2.2. The Concept of Successful Intelligence as an Integrated System

Crucially, Sternberg integrates these three components under the superordinate concept of successful intelligence, defined as the ability to formulate and achieve one’s personal and professional goals within a specific sociocultural context. This is accomplished by capitalizing on one’s strengths and by compensating for or correcting one’s weaknesses, using a balanced blend of analytical, creative, and practical abilities (Sternberg 1997). This definition is profoundly contextual and goal-oriented, shifting the focus from decontextualized academic aptitude to a more holistic and pragmatic understanding of human competence. It recognizes that “intelligent” behavior is contingent upon the environment and the individual’s aspirations. For an international student, success is not merely achieving a high grade; it is about adapting to a new culture, building a professional network, and ultimately achieving the personal and career goals that motivated their arduous educational journey. The three intelligences are not independent but work in a synergistic system. Analytical skills are necessary to evaluate the quality of creative ideas and to assess the effectiveness of practical actions. Creative skills are needed to generate solutions to problems that analysis has identified. Practical skills are required to implement creative ideas and to persuade others of their value.

### 2.3. The Evolution to Adaptive Intelligence and Its Ethical Dimension

In his more recent work, Sternberg (2019, 2021a) has expanded the concept of successful intelligence into a broader theory of “adaptive intelligence,” placing greater emphasis on the application of intelligence to solve complex, real-world problems for the *common good*. This evolution reflects a growing concern that traditional notions of intelligence, often valorized in academia, have failed to adequately address pressing global challenges, from climate change to social inequality (Sternberg 2021a). Adaptive intelligence is defined as the set of skills and attitudes required to adapt to current challenges, anticipate future problems, and actively shape the environment to create a better, more ethical world for oneself and for future generations (Sternberg 2019).

This framework posits that analytical, creative, and practical abilities are fundamentally in the service of positive environmental adaptation and the pursuit of a common good. It conceptualizes intelligence as comprising not only cognitive abilities but also crucial dispositions, such as intellectual humility, active open-mindedness, and the willingness to take sensible risks and overcome obstacles in pursuit of meaningful goals (Sternberg 2021b). This shift reinforces the need for an educational model that prepares students to solve the ill-defined, complex, and high-stakes problems they will face beyond the classroom. For international students, this means developing not just the ability to write a critical essay, but the resilience, resourcefulness, and ethical orientation to thrive in a new environment while becoming responsible global citizens. It reframes academic success not as an end in itself, but as preparation for making a positive contribution to the world.

### 2.4. Critiques and Scholarly Reception of the Triarchic Model

However, the triarchic theory has not been without its critics, and a balanced review requires acknowledging the scholarly debates surrounding its claims. A primary critique centers on the empirical distinctiveness of the three intelligences, particularly practical intelligence, from general intelligence (Gottfredson 2003). While some studies have supported the independence of these constructs (Sternberg et al. 2001), others have found moderate to high correlations, suggesting they may not be entirely separate abilities (Brody 2003). Furthermore, scholars have raised concerns about the psychometric challenges of reliably measuring creative and practical intelligence, as they are often context-dependent and less amenable to standardized testing than analytical abilities (Deary and Sternberg 2021). These critiques do not invalidate the theory’s pedagogical utility but highlight the importance of viewing it as a heuristic framework for curriculum design rather than a definitive statement on cognitive structure.

## 3. Broadening the Theoretical Lens: Complementary Perspectives on Academic Competence

While Sternberg’s model provides a valuable tripartite structure, relying on it exclusively risks theoretical narrowness. A more robust pedagogical framework can be constructed by integrating insights from other influential theories that conceptualize academic competence not just as a cognitive attribute but as a pluralistic and socially situated phenomenon.

Shifting the focus from individual cognitive abilities to the social context of learning, Sociocultural Theory (SCT), rooted in the work of Vygotsky (1978), offers another crucial lens. SCT posits that learning is not an isolated mental process but a social one, mediated by language and other cultural tools within a “community of practice” (Lantolf et al. 2020). From this perspective, academic success is less about possessing a certain type of intelligence and more about successful socialization into the specific discourse and practices of an academic community (Duff 2010).

This view is powerfully articulated in the Academic Literacies model (Lea and Street 2006). This model contrasts with a “study skills” approach (which sees academic competence as a set of generic, transferable skills) and a “discourse socialization” approach (which focuses on acculturating students into disciplinary norms). Instead, the Academic Literacies model views academic work as a set of complex, context-specific social practices that are shaped by institutional power relations and issues of identity (Lillis and Tuck 2016). It helps explain why international students often struggle with the “hidden curriculum”—the unspoken rules, values, and expectations of their new academic environment. For EAP pedagogy, this perspective suggests that instruction must go beyond teaching linguistic forms and analytical skills to making the tacit social and cultural rules of academic practice explicit (Cao and Hu 2024). By integrating insights from SCT and Academic Literacies, the Triarchic EAP Model can be grounded in a social understanding of learning, framing practical intelligence not just as an individual skill but as the ability to successfully navigate and participate in a new community of practice.

## 4. A Triarchic and Sociocultural Critique of EAP Pedagogy

Applying this broadened theoretical lens to the field of English for Academic Purposes reveals a pedagogical landscape of pronounced strengths and significant, often unacknowledged, gaps. While EAP has developed a sophisticated methodology for one facet of intelligence, the others remain comparatively marginalized, resulting in an imbalanced preparation for the realities of academic life.

### 4.1. The Dominance of Analytical Intelligence in EAP Curricula

Decades of research and practice have solidified a robust EAP pedagogy for cultivating analytical intelligence. The field’s very foundations are built on analytical approaches to understanding academic language and skills. Needs analysis, a systematic investigation of learners’ target situations and the competencies required to succeed in them, ensures that EAP curricula are goal-directed and relevant (Hutchinson and Waters 1987; Long 2005). This is often paired with genre analysis, which provides students with an explicit understanding of the rhetorical structures, linguistic features, and communicative purposes of specific academic texts, such as research articles, essays, and case studies (Swales 1990; Hyland 2022).

By deconstructing genres into identifiable “moves” and “steps,” as famously demonstrated in Swales’ (1990) Create-A-Research-Space (CARS) model for research article introductions, instructors make the logic of academic communication transparent and learnable. These analytical frameworks are typically applied to teaching source-based writing, the cornerstone of most EAP writing courses. This pedagogy requires students to read, synthesize, and critically evaluate multiple scholarly sources to construct their own arguments (McCray and Hanks 2023; Merkel 2019). Such tasks directly engage and develop the skills of analysis, comparison, evaluation, and logical argumentation that are the hallmark of analytical intelligence as defined in Sternberg’s componential sub-theory. This established and well-researched pedagogical focus on analytical skills forms a solid foundation upon which a more comprehensive, triarchic model can and should be built. It is the field’s greatest strength, but its dominance has created an unbalanced curriculum that underprepares students for other critical aspects of academic functioning (Ding and Bruce 2017).

### 4.2. Fostering Creative Intelligence: An Underdeveloped Potential

While analytical skills are explicitly taught and assessed, creative intelligence is often addressed only implicitly, if at all. The primary site for creative academic work, particularly at the graduate level, is the formulation of an original research project, which is typically articulated in a research proposal. This genre requires students to move beyond summarizing existing knowledge to identifying a gap and formulating a novel question or hypothesis (Flowerdew and Forest 2015). However, research proposals are frequently what Swales (1990) termed an “occluded genre,” meaning their conventions and prototypical examples are largely hidden from public view and not formally taught, making them exceedingly difficult for students—especially those from different academic cultures—to master without explicit guidance (Cadman 2002; Swales 1990).

A student may possess a highly innovative idea but lack the specific genre knowledge to frame it persuasively within the expected rhetorical structure. This gap between idea generation (a core creative act) and its formal articulation stifles creativity, as students struggle with the tacit expectations of how to establish a research space, articulate a problem, and justify the significance of their proposed contribution. Fostering creative intelligence in EAP thus requires more than generic brainstorming exercises. It demands a pedagogical approach that systematically demystifies occluded genres through explicit, genre-based instruction (Swales 1990). Furthermore, creativity in academia is not solely about grand, paradigm-shifting ideas. It also involves cognitive flexibility, such as the ability to adapt to unfamiliar academic tasks or to synthesize disparate ideas into a new theoretical framework (Corazza 2016). The strategic use of generative AI, for instance, can serve as a dialogic partner to help students brainstorm and refine novel ideas (Miao and Zhang 2024), though the development of critical AI literacy itself represents a key practical skill. Without a deliberate pedagogical focus, creative potential remains underdeveloped, leaving students ill-equipped for the generative and innovative aspects of scholarship.

### 4.3. The Neglect of Practical Intelligence: Tacit Knowledge and Pragmatic Competence, and Digital Literacy

Practical intelligence remains the most marginalized competency in mainstream EAP pedagogy (see Hyland 2022; Lillis and Tuck 2016 for discussions of EAP’s traditional focus). While certain skills like email etiquette may be addressed, they are often treated as isolated, peripheral “tips” rather than as manifestations of a core intellectual ability grounded in sociocultural understanding and the application of tacit knowledge. This neglect is particularly detrimental as it fails to address the need to navigate the complex social practices and ‘hidden curriculum’ that the Academic Literacies model identifies as central to academic success (Lea and Street 2006).

Pragmatic competence—the ability to use language appropriately in social contexts to achieve communicative goals—is essential for navigating the complex social world of the university (Taguchi 2019). A significant body of research on academic communication, particularly email requests, reveals that international students often struggle with conventions of directness, politeness, mitigation, and register when communicating with faculty and administrative staff (Chen 2015). These pragmatic difficulties stem from a lack of both sociopragmatic knowledge (understanding the social conventions and power dynamics of the context) and pragmalinguistic knowledge (knowing the specific linguistic forms to realize a communicative act appropriately) (Thomas 1983). Such “pragmatic failure” can lead to miscommunication, negative impressions, and strained student-faculty relationships, thereby hindering access to crucial academic support, mentorship, and research opportunities (Biesenbach-Lucas 2007).

Beyond specific communicative acts, practical intelligence is fundamental to the entire process of sociocultural adaptation. The ability to understand unspoken social norms, build effective support networks, and navigate the “hidden curriculum” of academic departments is a direct application of practical intelligence—the acquisition and use of tacit knowledge (Searle and Ward 1990; Ward and Kennedy 1999). This knowledge includes understanding departmental politics, interpreting the implicit expectations of an assignment, knowing when and how to participate in class, and managing relationships with peers and supervisors (Austin 2002; Duff 2010). Success in these areas is essential for student retention, well-being, and overall academic achievement (Tinto 1994).

Furthermore, in the current academic landscape, practical intelligence must also encompass digital literacy, particularly the critical and ethical use of generative AI. The ability to leverage tools like ChatGPT (based on the GPT-4 model; OpenAI) as a “research assistant” or “dialogic partner” for tasks such as exploring literature or refining arguments, while simultaneously understanding their limitations, biases, and the imperative to avoid plagiarism, is a crucial 21st-century academic skill (Guo and Wang 2024; Pack and Maloney 2023). This requires careful pedagogical design to ensure that AI is used to augment, rather than replace, students’ own critical processes (Nelson et al. 2025). By failing to systematically teach and assess skills related to pragmatic competence, sociocultural navigation, and critical AI literacy, EAP programs leave students to acquire this crucial form of intelligence through stressful, inefficient, and often unsuccessful trial and error.

## 5. The Triarchic EAP Model: A Proposed Conceptual Framework

To address the limitations of conventional EAP pedagogy and the imbalanced development of student competencies, this paper proposes the Triarchic EAP Model. This is a comprehensive framework designed to systematically and concurrently develop students’ analytical, creative, and practical intelligence. The model’s central aim is to move beyond mere linguistic preparation and instead foster the holistic competencies required for successful academic socialization and long-term achievement.

### 5.1. Core Principles: Integration, Scaffolding, and Transferability

**Figure 2:**
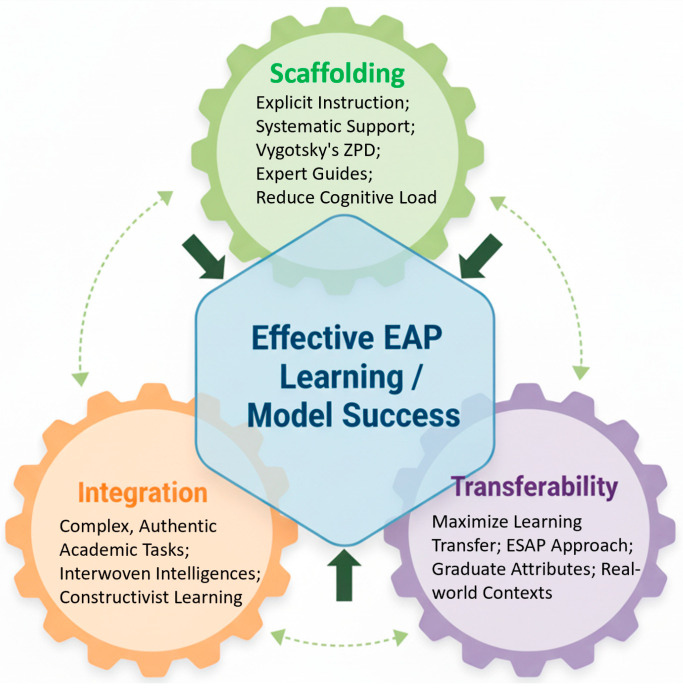
The Interplay of Pedagogical Principles.

The model is guided by three core pedagogical principles derived from established educational and sociocultural theories of learning:

1. Integration: The three intelligences are not conceptualized or taught as discrete, isolated units. Instead, they are interwoven within complex, authentic academic tasks. For example, a single capstone project might require students to analytically evaluate a body of literature, creatively synthesize it to propose a novel research question, and practically communicate their ideas in a simulated conference presentation and a subsequent follow-up email to a fictional professor. This principle reflects the reality of academic work, where analysis, creativity, and practical application are rarely separated. It aligns with constructivist learning theories that emphasize learning through complex, realistic problem-solving (Biggs and Tang 2011).
2. Scaffolding: Recognizing the cognitive and linguistic complexity of these integrated tasks, the model emphasizes the necessity of explicit instruction and systematic scaffolding, grounded in Vygotsky’s (1978) sociocultural theory of learning. Instructors act as expert guides, providing students with the necessary genre knowledge, linguistic tools, strategic frameworks, and cultural insights before they are expected to perform independently. This approach makes the tacit expectations and hidden rules of academia visible and manageable, reducing cognitive load and empowering students to tackle challenging tasks with growing confidence and autonomy (Hammond and Gibbons 2005).
3. Transferability: The curriculum is explicitly designed to maximize the transfer of learning from the EAP classroom to other academic, professional, and social contexts (Perkins and Salomon 1992). This is achieved through two primary mechanisms. First, by explicitly connecting EAP tasks to the demands of students’ specific disciplines (an English for Specific Academic Purposes, or ESAP, approach), ensuring immediate relevance. Second, by framing the skills learned within the broader context of institutional graduate attributes (e.g., “critical thinking,” “innovation,” “global citizenship”), making their value and applicability clear to students, faculty, and administrators (Barrie 2004; James 2014). This focus on transfer transforms EAP from a preliminary hurdle into a foundational component of a student’s entire educational trajectory. The interplay of these principles is visualized in Figure 2.

### 5.2. A Framework for Curriculum Design and Assessment

The operationalization of the Triarchic EAP Model is detailed in Table 1. This framework provides a practical guide for curriculum designers and practitioners, outlining key learning objectives, sample pedagogical activities, proposed assessment methods, and measurable indicators for each form of intelligence. It offers concrete, evidence-based methods for teaching and, crucially, for assessing the often-neglected creative and practical dimensions of academic competence, thereby ensuring that all three intelligences are treated with equal pedagogical seriousness.

## 6. Implications and Future Directions

The adoption of the Triarchic EAP Model has the potential to carry profound implications for pedagogy, institutional policy, and future research. It calls for a fundamental shift in how EAP is conceptualized and delivered, moving it from the periphery to the core of the international student experience and positioning it as a central driver of holistic intellectual development.

### 6.1. Pedagogical and Professional Development Implications

The model necessitates a significant evolution in the role of the EAP instructor, from a language teacher primarily concerned with grammar and rhetoric to a facilitator of holistic academic acculturation. This expanded role requires a pedagogical toolkit capable of fostering creativity, developing pragmatic competence, and making the tacit rules of academia explicit (Pecorari 2006). This challenges the traditional separation of language, culture, and cognition, advocating for an integrated approach where learning a new academic discourse is understood as a form of cognitive and social apprenticeship into a community of practice (Lave and Wenger 1991; Wenger-Trayner and Wenger-Trayner 2020).

Consequently, there is a pressing need for professional development programs that equip EAP instructors with the theoretical knowledge and practical strategies to teach and assess these broader intellectual skills. This includes training in creativity pedagogy (e.g., problem-based learning), the principles of second language pragmatics instruction, and intercultural communication (Ren et al. 2023; Turner 2011). Such training would empower instructors to guide students in becoming not just proficient writers, but also resourceful, adaptable, and confident members of their new academic communities.

### 6.2. Institutional and Policy Implications

For the model to be effective, it requires robust institutional support and a strategic shift in how EAP programs are perceived and positioned within the university. The principle of transferability is contingent upon greater and more systematic collaboration between EAP units and disciplinary faculties (Benesch 2001; Hyland 2022). Such partnerships, which could include co-teaching, shared curriculum development, or the joint creation of assessment rubrics, are essential to ensure that the skills taught in EAP are directly relevant to the specific genres and tasks of students’ chosen fields, thereby creating a supportive “transfer climate” (James 2014).

Furthermore, universities should formally recognize the central role EAP programs can play in developing the competencies outlined in their institutional graduate attribute frameworks (Guzmán-Valenzuela et al. 2021; Murphy et al. 2023). Many universities have established ambitious frameworks of “Graduate Attributes”—such as critical thinking, creativity, communication skills, and global citizenship. However, these attributes are often difficult to embed and assess systematically within content-focused disciplinary courses (Chan et al. 2017). The Triarchic EAP Model, by explicitly mapping its learning objectives onto these attributes, positions the EAP classroom as a unique and intentional site for their development and assessment. By embracing this model, universities can leverage their EAP programs not as remedial services for students with linguistic deficits, but as integral, high-impact hubs for cultivating the holistic, transferable skills that define a 21st-century graduate.

### 6.3. Limitations of the Proposed Model

As a conceptual framework, the Triarchic EAP Model has several limitations that must be acknowledged.

First, its implementation requires a substantial investment in instructor training and curriculum redesign, which may pose challenges for institutions with limited resources. Potential Solution: A phased professional development model could be adopted, starting with collaborative workshops focused on designing and assessing tasks for one non-analytical intelligence (e.g., practical communication scenarios), allowing for gradual, sustainable implementation.

Second, the assessment of creative and practical intelligence is inherently more complex and subjective than the assessment of analytical skills. Potential Solution: This can be mitigated through the collaborative development of shared, context-specific rubrics between EAP units and disciplinary faculty, ensuring that assessment criteria are both reliable and relevant to students’ fields of study (Daumiller and Meyer 2025). Furthermore, adopting portfolio-based assessment can capture developmental growth over time more effectively than single-point assessments.

Third, the model’s effectiveness is contingent on factors outside the EAP classroom, such as the degree of collaboration from disciplinary faculty and the broader institutional climate. Potential Solution: Piloting the model within a single supportive department or faculty can create a proof-of-concept and generate data-driven evidence of its value, which can then be used to advocate for wider institutional adoption.

Finally, the model is presented as a general framework, and its specific application will require considerable adaptation to different institutional contexts, student populations, and disciplinary norms. Its applicability beyond the Anglo-Western contexts from which much of the cited research derives has yet to be established.

### 6.4. An Agenda for Future Research

The Triarchic EAP Model is conceptual and requires empirical validation through rigorous research. A robust research agenda is needed to test, refine, and expand upon the framework. Key areas for investigation include:

Longitudinal impact studies are required to track the academic, professional, and personal trajectories of students who have participated in a Triarchic EAP curriculum. Such studies, employing mixed-methods designs, could measure long-term outcomes related to academic achievement (e.g., GPA, time to completion), publication rates, career progression, and psychological well-being, comparing these outcomes to those of control groups from traditional EAP programs.

A critical priority is the development and validation of assessment tools, such as robust rubrics and standardized scenarios, to reliably measure growth in creative and practical competencies within EAP contexts. This research should build on existing work in performance-based assessment to create instruments that are both valid and practical for classroom use.

Cross-contextual implementation research is necessary to investigate how the framework can be best adapted to meet the needs of students in different fields (e.g., STEM vs. humanities), at different academic levels (undergraduate vs. graduate), and in diverse higher education systems globally, particularly in non-Anglophone settings.

Further investigation into the role of technology is warranted, particularly exploring how digital tools, including generative AI, can be ethically and effectively integrated into the model. Research should focus on developing pedagogical strategies that use AI to augment, rather than replace, the development of students’ analytical, creative, and practical skills.

## 7. Conclusions

The increasing complexity and interconnectedness of global academia demand a reconceptualization of the skills required for student success. This review has argued that traditional EAP models, with their heavy emphasis on analytical intelligence, provide international students with only a partial toolkit for the challenges they face. By drawing on Sternberg’s theory of successful intelligence—critically examined and situated within a broader theoretical landscape that includes pluralistic and social views of learning—a more holistic and robust pedagogical vision emerges. The proposed Triarchic EAP Model offers a theoretically grounded and practically actionable framework for realizing this vision. It moves beyond a narrow focus on linguistic remediation to embrace the development of the whole student, fostering the analytical rigor to critique existing knowledge, the creative capacity to generate new knowledge, and the practical wisdom to apply that knowledge effectively and ethically in a complex world. By systematically integrating these three intelligences, the model positions the EAP classroom not as a preliminary, remedial stage, but as a dynamic and central environment where students are socialized into the multifaceted intellectual and social life of the university.

It must be emphasized that the framework proposed here is conceptual; its efficacy and scalability can only be determined through the rigorous empirical research outlined in the preceding section. Ultimately, this approach aims to do more than improve academic performance; it seeks to empower international students to become confident, adaptable, and innovative scholars prepared to make a meaningful difference in their fields and in the world.

## Figures and Tables

**Table 1:** A Framework for Curriculum Design and Assessment within the Triarchic EAP Model.

Intelligence	Key Learning Objectives	Sample Pedagogical Activities & Tasks	Proposed Assessment Methods	Measurable Indicators/Evidence	Alignment with Graduate Attributes
Analytical	- Critically evaluate and synthesize information from multiple scholarly sources. - Construct coherent, evidence-based academic arguments. - Deconstruct rhetorical structures and identify underlying assumptions in texts.	- *Source-Based Synthesis Essay:* Students integrate multiple scholarly sources to support a nuanced thesis (Merkel 2019). - *Critical Reading Journal:* Students analyze and respond to weekly readings, focusing on argumentation, evidence, and authorial stance. - *Annotated Bibliography:* Students summarize, analyze, and evaluate the relevance and credibility of sources.	- *Analytic Rubrics:* Assessing discrete criteria such as thesis clarity, source integration, argumentation, and critical depth (Andrade 2005). - *Structured Peer Review:* Students evaluate each other’s drafts using specific analytical criteria (Topping 2009).	- Score on a validated analytical writing rubric. - Quality of critical analysis in reading journal entries. - Demonstrated ability to identify logical fallacies in peer review tasks.	- Critical Thinking - Research and Inquiry Skills - Information Literacy
Creative	- Generate novel, significant, and viable research questions or topics. - Adapt rhetorical strategies for unfamiliar or “occluded” academic genres. - Propose innovative solutions or synthesize disparate perspectives.	- *Research Proposal Project:* A scaffolded project moving from literature gap analysis to a fully formed proposal. - *“Problem-Posing” Workshops:* Students collaboratively identify gaps and contradictions in readings to formulate researchable questions. - *Genre Transformation Task:* Students adapt a research article summary into a different format (e.g., a three-minute thesis script, a conference poster).	- *Creativity Rubrics:* Assessing originality, feasibility, clarity of contribution, and persuasiveness of the research idea (Vaezi and Rezaei 2019). - *Portfolio of Ideas:* A developmental collection of brainstormed research questions, assessed for growth and refinement. - *Reflective Statement:* Students articulate and justify the innovative contribution of their proposed research.	- Originality and feasibility scores on a research proposal rubric. - Number and quality of viable research questions generated in workshops. - Successful rhetorical adaptation demonstrated in genre transformation tasks.	- Innovation and Creativity - Problem-Solving - Adaptability & Flexibility
Practical	- Demonstrate pragmatic competence in key academic communicative acts. - Navigate the sociocultural norms and “hidden curriculum” of the academic community. - Apply tacit knowledge and critical AI literacy effectively.	- *Academic Email Simulation:* Using role-play, students respond to realistic email scenarios (e.g., requesting an extension, clarifying a grade) (Biesenbach-Lucas 2007). - *Case Studies in Academic Culture:* Students analyze scenarios involving sociocultural challenges (e.g., plagiarism, classroom participation norms) (Odena and Burgess 2017). - *Ethical AI Use Workshop:* Students use AI to perform a task, then critique the output and discuss the ethical implications of its use.	- *Portfolio Assessment:* A collection of student-written emails and other communications, assessed for pragmatic appropriateness (Ishihara and Cohen 2021). - *Performance-Based Scenarios:* Role-playing assessments of students’ ability to handle difficult academic conversations. - *Reflective Journals:* Students document and analyze their sociocultural adaptation challenges and successes (Duff 2010).	- Reduced pragmatic errors in email simulations as measured by a validated rubric. - Performance on standardized scenario-based tests of pragmatic competence. - Quality of metacognitive reflection in journals on sociocultural adaptation.	- Effective Communication - Global & Cultural Competence - Professionalism & Ethical Conduct

## Data Availability

No new data were created or analyzed in this study. Data sharing is not applicable to this article.

## Abbreviations

The following abbreviations are used in this manuscript:

EAP	English for Academic Purposes
CARS	Create-A-Research-Space

## References

[B1-jintelligence-13-00134] Alharbi Eman, Smith Andrew (2018). Review of the literature on stress and wellbeing of international students in English-speaking countries. International Education Studies.

[B2-jintelligence-13-00134] Andrade Heidi Goodrich (2005). Teaching with rubrics: The good, the bad, and the ugly. College Teaching.

[B3-jintelligence-13-00134] Austin Ann E. (2002). Preparing the next generation of faculty: Graduate school as socialization to the academic career. The Journal of Higher Education.

[B4-jintelligence-13-00134] Barrie Simon C. (2004). A research-based approach to generic graduate attributes policy. Higher Education Research & Development.

[B5-jintelligence-13-00134] Benesch Sarah (2001). Critical English for Academic Purposes: Theory, Politics, and Practice.

[B6-jintelligence-13-00134] Biesenbach-Lucas Sigrun (2007). Students writing emails to faculty: An examination of E-politeness among native and non-native speakers of English. Language Learning & Technology.

[B7-jintelligence-13-00134] Biggs John, Tang Catherine (2011). Teaching for Quality Learning at University.

[B8-jintelligence-13-00134] Brody Nathan (2003). Construct validation of the Sternberg Triarchic Abilities Test: Comment and reanalysis. Intelligence.

[B9-jintelligence-13-00134] Cadman Kate (2002). English for academic possibilities: The research proposal as a contested site in postgraduate genre pedagogy. Journal of English for Academic Purposes.

[B10-jintelligence-13-00134] Cao Feng, Hu Guangwei (2024). English for Academic Purposes. Reference Module in Social Sciences.

[B11-jintelligence-13-00134] Chan Cecilia K.Y., Fong Emily T.Y., Luk Lillian Y.Y., Ho Robbie (2017). A review of literature on challenges in the development and implementation of generic competencies in higher education curriculum. International Journal of Educational Development.

[B12-jintelligence-13-00134] Chen Yuan-shan (2015). Chinese learners’ cognitive processes in writing email requests to faculty. System.

[B13-jintelligence-13-00134] Corazza Giovanni Emanuele (2016). Potential originality and effectiveness: The dynamic definition of creativity. Creativity Research Journal.

[B14-jintelligence-13-00134] Cotton Debby R. E., Cotton Peter A., Shipway J. Reuben (2024). Chatting and cheating: Ensuring academic integrity in the era of ChatGPT. Innovations in Education and Teaching International.

[B15-jintelligence-13-00134] Daumiller Martin, Meyer Jennifer (2025). Advancing feedback research in educational psychology: Insights into feedback processes and determinants of effectiveness. Contemporary Educational Psychology.

[B16-jintelligence-13-00134] Deary Ian J., Sternberg Robert J. (2021). Ian Deary and Robert Sternberg answer five self-inflicted questions about human intelligence. Intelligence.

[B17-jintelligence-13-00134] de Wit Hans, Altbach Philip G. (2021). Internationalization in higher education: Global trends and recommendations for its future. Policy Reviews in Higher Education.

[B18-jintelligence-13-00134] Ding Alex, Bruce Ian (2017). The English for Academic Purposes Practitioner: Operating on the Edge of Academia.

[B19-jintelligence-13-00134] Duff Patricia A. (2010). Language socialization into academic discourse communities. Annual Review of Applied Linguistics.

[B20-jintelligence-13-00134] Flowerdew John, Forest Richard W. (2015). Signalling Nouns in English: A Corpus-Based Discourse Approach.

[B21-jintelligence-13-00134] Glass Chris R., Westmont Christina M. (2014). Comparative effects of belongingness on the academic success and cross-cultural interactions of domestic and international students. International Journal of Intercultural Relations.

[B22-jintelligence-13-00134] Gottfredson Linda S. (2003). Dissecting practical intelligence theory: Its claims and evidence. Intelligence.

[B24-jintelligence-13-00134] Gu Qing, Schweisfurth Michele, Day Christopher (2010). Learning and growing in a ‘foreign’ context: Intercultural experiences of international students. Compare: A Journal of Comparative and International Education.

[B23-jintelligence-13-00134] Guo Kai, Wang Deliang (2024). To resist it or to embrace it? Examining ChatGPT’s potential to support teacher feedback in EFL writing. Education and Information Technologies.

[B25-jintelligence-13-00134] Guzmán-Valenzuela Carolina, Gómez-González Carolina, Tagle Andrés Rojas-Murphy, Lorca-Vyhmeister Alejandro (2021). Learning analytics in higher education: A preponderance of analytics but very little learning?. International Journal of Educational Technology in Higher Education.

[B26-jintelligence-13-00134] Hammond Jenny, Gibbons Pauline (2005). Putting scaffolding to work: The contribution of scaffolding in articulating ESL education. Prospect.

[B27-jintelligence-13-00134] Hutchinson Tom, Waters Alan (1987). English for Specific Purposes: A Learning-Centred Approach.

[B28-jintelligence-13-00134] Hyland Ken (2022). English for Academic Purposes: A Research-Based Introduction.

[B29-jintelligence-13-00134] Hyland Ken, Jiang Feng (Kevin) (2021). Delivering relevance: The emergence of ESP as a discipline. English for Specific Purposes.

[B30-jintelligence-13-00134] Ishihara Noriko, Cohen Andrew D. (2021). Teaching and Learning Pragmatics: Where Language and Culture Meet.

[B31-jintelligence-13-00134] Jackson Denise (2015). Employability skill development in work-integrated learning: Barriers and best practice. Studies in Higher Education.

[B32-jintelligence-13-00134] James Mark A. (2014). Learning transfer in English-for-academic-purposes contexts: A systematic review of research. Journal of English for Academic Purposes.

[B33-jintelligence-13-00134] Kalsum Ummi, Ampa Andi Tenri, Hamid Radiah (2023). Implementation of integrated language skills in English teaching process. International Journal of Social Science and Education Research Studies.

[B34-jintelligence-13-00134] Lantolf James P., Thorne Steven L., Poehner Matthew E., VanPatten Bill, Keating Gregory D., Wulff Stefanie (2020). Sociocultural theory and second language development. Theories in Second Language Acquisition: An Introduction.

[B35-jintelligence-13-00134] Lave Jean, Wenger Etienne (1991). Situated Learning: Legitimate Peripheral Participation.

[B36-jintelligence-13-00134] Lea Mary R., Street Brian V. (2006). The “Academic Literacies” model: Theory and applications. Theory Into Practice.

[B37-jintelligence-13-00134] Lillis Theresa, Tuck Jackie, Hyland Ken, Shaw Philip (2016). Academic literacies: A critical perspective. The Routledge Handbook of English for Academic Purposes.

[B38-jintelligence-13-00134] Long Michael H. (2005). Second Language Needs Analysis.

[B39-jintelligence-13-00134] Luo Man, Ikram Awan Hasham, Zhang Xiaofang, Peng Fang, Zhao Jing, Deng Haijun (2024). Role of attachment style, acculturation orientation, and social support in the acculturation of international students in China. International Journal of Intercultural Relations.

[B40-jintelligence-13-00134] Maharaj Reshin, Ndwiga Dorothy, Chutiyami Muhammad (2024). Mental health and wellbeing of international students in Australia: A systematic review. Journal of Mental Health.

[B41-jintelligence-13-00134] Mao Yuezu (2024). Academic adaptation of international students in the Chinese higher education environment: A case study with mixed methods. International Journal of Intercultural Relations.

[B42-jintelligence-13-00134] McCray Diana, Hanks Judith (2023). Learners’ perceptions of writing difficulties on a pre-sessional EAP programme in a British university. Journal of Academic Writing.

[B43-jintelligence-13-00134] Merkel Warren (2019). A case study of undergraduate L2 writers’ concerns with source-based writing and plagiarism. TESOL Journal.

[B44-jintelligence-13-00134] Miao Chenglong, Zhang Shuai (2024). The cross-cultural adaptation of Chinese international students: An empirical study on sequential-mediated effects. Frontiers in Psychology.

[B45-jintelligence-13-00134] Murphy Mike, Coleman Adel, Donoghue Eleanor, Hunt Eithne (2023). Development of the UCC Graduate Attributes and Values Compass (GAP Compass). Paper presented at the European Learning & Teaching Forum.

[B46-jintelligence-13-00134] Nelson Andrew S., Santamaría Paola V., Javens Josephine S., Ricaurte Marvin (2025). Students’ Perceptions of Generative Artificial Intelligence (GenAI) Use in Academic Writing in English as a Foreign Language. Education Sciences.

[B47-jintelligence-13-00134] Ng Ting Kin, Wang Kitty Wan Ching, Chan Wai (2017). Acculturation and cross-cultural adaptation: The moderating role of social support. International Journal of Intercultural Relations.

[B48-jintelligence-13-00134] Odena Oscar, Burgess Hilary (2017). How doctoral students and graduates describe facilitating experiences and strategies for their thesis writing learning process: A qualitative approach. Studies in Higher Education.

[B49-jintelligence-13-00134] Pack Austin, Maloney Jeffrey (2023). Using generative artificial intelligence for language education research: Insights from using OpenAI’s ChatGPT. TESOL Quarterly.

[B50-jintelligence-13-00134] Pecorari Diane (2006). Visible and occluded citation features in postgraduate second-language writing. English for Specific Purposes.

[B51-jintelligence-13-00134] Perkins David N., Salomon Gavriel, Husen Torsten, Postlethwaite T. Neville (1992). Transfer of Learning. The International Encyclopedia of Education.

[B52-jintelligence-13-00134] Razgulin Jevgenij, Argustaitė-Zailskienė Gita, Šmigelskas Kastytis (2023). The role of social support and sociocultural adjustment for international students’ mental health. Scientific Reports.

[B53-jintelligence-13-00134] Ren Wei, Li Shaofeng, Lü Xiaoxuan (2023). A meta-analysis of the effectiveness of second language pragmatics instruction. Applied Linguistics.

[B54-jintelligence-13-00134] Ros Timothy, Samuel Anita (2024). Navigating the AI frontier: A guide for ethical academic writing. eLearn.

[B55-jintelligence-13-00134] Ryan Janette (2011). Teaching and learning for international students: Towards a transcultural approach. Teachers and Teaching.

[B56-jintelligence-13-00134] Searle Wendy, Ward Colleen (1990). The prediction of psychological and sociocultural adjustment during cross-cultural transitions. International Journal of Intercultural Relations.

[B57-jintelligence-13-00134] Sheng Laping, Dai Junxia, Lei Jinhuo (2022). The impacts of academic adaptation on psychological and sociocultural adaptation among international students in China: The moderating role of friendship. International Journal of Intercultural Relations.

[B58-jintelligence-13-00134] Sternberg Robert J. (1985). Beyond IQ: A Triarchic Theory of Human Intelligence.

[B59-jintelligence-13-00134] Sternberg Robert J. (1997). Successful Intelligence: How Practical and Creative Intelligence Determine Success in Life.

[B60-jintelligence-13-00134] Sternberg Robert J. (2003). What is an ‘expert student?’. Educational Researcher.

[B61-jintelligence-13-00134] Sternberg Robert J., Sternberg Robert J., Kaufman Scott Barry (2011). The theory of successful intelligence. The Cambridge Handbook of Intelligence.

[B62-jintelligence-13-00134] Sternberg Robert J. (2019). A theory of adaptive intelligence and its relation to general intelligence. Journal of Intelligence.

[B63-jintelligence-13-00134] Sternberg Robert J. (2021a). Adaptive intelligence: Its nature and implications for education. Education Sciences.

[B64-jintelligence-13-00134] Sternberg Robert J. (2021b). Adaptive Intelligence: Surviving and Thriving in Times of Uncertainty.

[B65-jintelligence-13-00134] Sternberg Robert J., Nokes Catherine, Geissler Paul Wenzel, Prince Ruth, Okatcha Frederick, Bundy Donald A., Grigorenko Elena L. (2001). The relationship between academic and practical intelligence: A case study in Kenya. Intelligence.

[B66-jintelligence-13-00134] Sternberg Robert J., Forsythe George B., Hedlund Jennifer, Horvath Joseph A., Wagner Richard K., Williams Wendy M., Snook Scott A., Grigorenko Elena L. (2000). Practical Intelligence in Everyday Life.

[B67-jintelligence-13-00134] Swales John M. (1990). Genre Analysis: English in Academic and Research Settings.

[B68-jintelligence-13-00134] Taguchi Naoko (2019). The Routledge Handbook of Second Language Acquisition and Pragmatics.

[B69-jintelligence-13-00134] Thomas Jenny (1983). Cross-cultural pragmatic failure. Applied Linguistics.

[B70-jintelligence-13-00134] Tinto Vincent (1994). Leaving College: Rethinking the Causes and Cures of Student Attrition.

[B71-jintelligence-13-00134] Topping Keith J. (2009). Peer assessment. Theory into Practice.

[B72-jintelligence-13-00134] Turner Joan (2011). Language in the Academy: Cultural Reflexivity and Intercultural Dynamics.

[B73-jintelligence-13-00134] Vaezi Maryam, Rezaei Saeed (2019). Development of a rubric for evaluating creative writing: A multi-phase research. New Writing.

[B74-jintelligence-13-00134] Vygotsky Lev Semyonovich (1978). Mind in Society: The Development of Higher Psychological Processes.

[B75-jintelligence-13-00134] Wagner Richard K., Sternberg Robert J. (1987). Tacit knowledge in managerial success. Journal of Business and Psychology.

[B76-jintelligence-13-00134] Ward Colleen, Kennedy Antony (1999). The measurement of sociocultural adaptation. International Journal of Intercultural Relations.

[B77-jintelligence-13-00134] Wei Xiaofang (2025). Sociocultural adaptation of Chinese international students in the United States and its influencing factors. Frontiers in Psychology.

[B78-jintelligence-13-00134] Wenger-Trayner Etienne, Wenger-Trayner Beverly (2020). Learning to Make a Difference: Value Creation in Social Learning Spaces.

[B79-jintelligence-13-00134] Yang Peidong (2022). Rethinking international student mobility through the lens of “crisis” at a juncture of pandemic and global uncertainties. Asia Pacific Journal of Education.

[B80-jintelligence-13-00134] Yin Zizhuo, Ong Lee Za, Qiao Ming (2024). Psychological factors associated with Chinese international students’ well-being in the United States. Journal of International Students.

